# Fluke-Related Cholangiocarcinoma: Challenges and Opportunities

**DOI:** 10.3390/pathogens12121429

**Published:** 2023-12-08

**Authors:** J. Luis Espinoza

**Affiliations:** Faculty of Health Sciences, Kanazawa University, Kanazawa 920-0942, Ishikawa, Japan; luis@staff.kanazawa-u.ac.jp; Tel.: +76-265-2500; Fax: +76-234-4351

Cholangiocarcinoma encompasses a heterogeneous group of highly aggressive malignancies, arising from the biliary tract, that are often associated with poor survival rates. Depending on the anatomical regions in which these tumors develop, they are classified into intrahepatic cholangiocarcinoma, extrahepatic cholangiocarcinoma, and gallbladder cancer, with differences in epidemiology, pathogenesis, treatment, and prognosis. In addition, cholangiocarcinomas can be categorized into fluke-related cholangiocarcinomas and non-fluke-associated cholangiocarcinomas based on well-established epidemiological characteristics. In most Western countries, cholangiocarcinomas are relatively rare tumors (with an incidence of 1–2 per 100,000 people per year); although, their incidence has steadily increased over the past few decades. In these regions, where tumors are predominantly non-fluke-associated cholangiocarcinoma, conditions that promote chronic inflammation in the biliary tract, such as choledocholithiasis, cholelithiasis, primary sclerosing cholangitis, liver cirrhosis, non-alcoholic fatty liver disease, hepatitis B, obesity, and non-alcoholic fatty liver disease, appear to increase cholangiocarcinoma susceptibility [1].

Fluke-related cholangiocarcinomas, also known as epidemic cholangiocarcinomas, are particularly prevalent in certain regions of Asia, where parasitic liver flukes such as *Clonorchis sinensis*, *Opisthorchis viverrine*, and *Opisthorchis felineus* are endemic. Humans can be infected by these pathogens after consuming raw or undercooked fish containing the infective cysts (metacercariae) of the parasites. After hatching in the duodenum, the larval flukes migrate to the biliary tract, where the mature fluke reside. The presence of liver flukes in the bile duct leads to tissue damage, chronic inflammation, oxidative stress, and periductal fibrosis; changes that increase the risk of carcinogenesis. In addition, the persistent inflammatory response to these pathogens can be associated with DNA damage and epigenetic alterations in the biliary epithelial cells, which also contribute to increasing susceptibility to cholangiocarcinoma. 

Infection by *Clonorchis sinensis* predominates in certain areas of the Korean Peninsula, far-eastern Russia, mainland China, and Taiwan, while *Opisthorchis felineus* is prevalent in western Siberia and Kazakhstan. *Opisthorchis viverrine* infection, commonly referred to as opisthorchiasis, contributes to the vast majority of fluke-related cholangiocarcinomas and is highly prevalent in the lower Mekong River basin, affecting millions of people living in various regions of Thailand, Laos, Cambodia, Vietnam, and Myanmar [2]. 

Liau and colleagues have extensively reviewed the burden of *Opisthorchis viverrine* infection on populations living in areas of Southeast Asia in which these pathogens are highly prevalent. In addition to covering the key aspects of the epidemiology, parasitology, and health implications associated with *Opisthorchis viverrine* infection, the authors also highlighted the socioeconomic burden and public health impact of this neglected tropical disease. Their article emphasizes the need for comprehensive efforts involving prevention, early diagnosis, and continued surveillance to combat *Opisthorchis viverrine* infection. In particular, the article highlights the achievements of preventive interventions such as the Lawa model in Thailand, which integrates education and surveillance and has shown measurable success in reducing the infection rate, potentially serving as a model for other endemic regions [3]. In this regard, it is important to consider the critical role of combating parasite reinfection in all programs aimed at controlling the burden of *Opisthorchis viverrine* infection, as high reinfection rates have been reported among individuals living in endemic areas, with a reinfection rate of 15% in the two years following an intervention in the general population, and of 23% among fishermen [4]. 

Eradicating *Opisthorchis viverrini* infection involves the administration of anthelmintic drugs, with praziquantel being the first-line treatment. Tribendimidine is an alternative drug choice with comparable efficacy, and albendazole appears to show a similar efficacy when administered for five to seven days; although, the evidence available on the efficacy of albendazole is more limited [5]. Importantly, though evidence of the long-term effects of anthelmintic therapy in terms of preventing the development of fibrotic sequelae in the biliary tract is limited, an early study reported the regression of various biliary tract abnormalities associated with *Opisthorchis viverrini* infection following treatment with praziquantel [6]. Further studies are needed to delineate the role of parasite eradication to attenuate the long-term sequelae of chronic inflammation associated with parasite infection and ultimately prevent cholangiocarcinoma among high-risk individuals.

As is the case for various malignancies, immunotherapy combined with chemotherapy has emerged as a promising therapeutic strategy to treat cholangiocarcinoma. In addition, with advancements in the understanding of cholangiocarcinoma carcinogenesis, multiple targets for intervention, such as fibroblast growth factor receptor 2 (FGFR2), human epidermal growth factor receptor 2 (HER2), and high tumor mutation burden (TMB-H), have been identified, paving the way for the development of novel targeted therapies. Indeed, various agents have been approved for use as second-line therapies or are under evaluation as first-line therapeutics in the treatment of cholangiocarcinoma. Importantly, mutations in the genes encoding isocitrate dehydrogenase 1 (IDH1) and its analogous IDH2 are frequently detected in various cancers, including glioma (80%), chondrosarcoma (80%), and acute myeloid leukemia (20%). Mutated IDH enzymes lead to the production of D-2-hydroxyglutarate (2-HG), which promotes cancer cell survival and growth. The discovery that IDH mutations occur in up to 20% of patients with cholangiocarcinoma represents a breakthrough in targeted therapy for cholangiocarcinoma [7].Given the importance of IDH mutations in cholangiocarcinoma, in 2021, ivosidenib, a selective IDH-mutant inhibitor, was approved for use in adult patients with previously treated, locally advanced, or metastatic cholangiocarcinoma harboring an IDH1 mutation. Nevertheless, the frequency and clinical implications of IDH mutations in fluke-related cholangiocarcinoma remain unknown, and, to the best of our knowledge, no comparative studies on this issue have been published from populations living in endemic areas. Given the therapeutic potential of IDH-targeted therapies in various cancers, further studies are necessary to determine the role and clinical implications of these mutations in fluke-related cholangiocarcinoma. 

Similarly, studies aimed at delineating the expression of immunity suppressing molecules, such as cytotoxic T lymphocyte antigen 4 (CTLA4) and programmed cell death 1 (PD1) or its ligand PDL1, in fluke-related cholangiocarcinoma are also needed, as various immune checkpoint inhibitors are currently being tested in patients with cholangiocarcinoma, and recent clinical trials have shown that immunotherapy combined with standard chemotherapy has been demonstrated to have an acceptable safety and measurable clinical benefits in patients with cholangiocarcinoma [8].

Despite the fact that targeted therapies and immunotherapy can be translated into measurable progress in the efficacy of treatments and a drastic reduction in therapy-related toxicity, which together may offer the prospect of changing the treatment paradigm for fluke-related cholangiocarcinoma, several limitations have been associated with these novel therapies. In particular, data from recent clinical trials show that most targeted therapies and immunotherapy regimens result in only a few months of progression-free survival, and the improvement in overall survival is rather modest [8,9,10]. Furthermore, the elevated cost of novel therapeutic agents may be prohibitive for populations living in areas where fluke-related cholangiocarcinoma is endemic.
